# An augmented food strategy leads to complete energy compensation during a 15‐day military training expedition in the cold

**DOI:** 10.14814/phy2.14591

**Published:** 2021-05-31

**Authors:** Keyne Charlot, Didier Chapelot, Julien Siracusa, Chloé Lavoué, Philippe Colin, Pauline Oustric, David Thivel, Graham Finlayson, Cyprien Bourrilhon

**Affiliations:** ^1^ Unité de Physiologie de l'Exercice et des Activités en Conditions Extrêmes Département Environnements Opérationnels Institut de Recherche Biomédicale des Armées Bretigny‐Sur‐Orge France; ^2^ LBEPS Univ Evry IRBA Université Paris Saclay Evry France; ^3^ Centre de Recherche en Epidémiologie et Statistique Equipe de Recherche en Epidémiologie Nutritionnelle (EREN) Inserm (U1153) Inra (U1125) Cnam Université Paris 13 Bobigny France; ^4^ Appetite Control Energy Balance Research Group School of Psychology Faculty of Medicine and Health University of Leeds Leeds United Kingdom; ^5^ Laboratoire des adaptations Métaboliques à l’Exercice en conditions Physiologiques et Pathologiques (EA 3533) Université Clermont Auvergne Clermont‐Ferrand France

**Keywords:** arctic, energy compensation, energy deficiency, food preferences, military training, rations

## Abstract

Soldiers on military expeditions usually fail to compensate for the increase in energy expenditure, with potential deleterious consequences. We therefore analyzed the characteristics of energy compensation in 12 male soldiers, during a 15‐day expedition in the cold, while alleviating some of the contextual limitations of food intake (~20‐MJ daily bags of easy‐to‐use, highly palatable and familiar foods with multiple and long breaks allowed during the day). Body and fat mass losses were low and moderate, respectively (−1.13 ± 1.42% and −19.5 ± 15.6%, respectively, *p < *.021). Mean energy intake (EI) was high (~16.3 MJ) and increased at each third of the expedition (15.3 ± 2.1, 16.1 ± 2.1, and 17.6 ± 2.0 for D1–5, D6–10 and D11–15, respectively, *p < *.012). This resulted in reaching a neutral energy balance as soon as the D6 to 10 period and reaching normal energy availability during D11 to 15. Participants only increased their EI during the mid‐day (10:00–14:00) period (*p = *.002) whereas hunger and thirst only increased in the morning, with higher scores during D11–15 than D1–5 (*p < *.009). Last, the reward value of sweet foods was also higher during D11–15 than during D1–5 (*p = *.026). The changes in body mass were positively associated with EI (*r = *0.598, *p = *.040) and carbohydrate intake (*r = *0.622, *p = *.031). This study indicates that complete energy compensation can be reached in challenging field conditions when food intake is facilitated, offering some guidelines to limit energy deficit during operational missions.

## Graphical abstract

We analyzed the characteristics of energy compensation in 12 male soldiers, during a 15‐day expedition in the cold, while alleviating some of the contextual limitations of food intake (~20‐MJ daily bags of easy‐to‐use, highly palatable and familiar foods with multiple and long breaks allowed during the day). This study indicates that complete energy compensation can be reached in challenging field conditions when food intake is facilitated, offering some guidelines to limit energy deficit during operational missions.

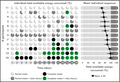

## 1 INTRODUCTION

Pioneer (Edholm, Fletcher, Widdowson, & Mccance, 1955; Mayer, Roy, & Mitra, 1956) and contemporary (Beaulieu, Hopkins, Blundell, & Finlayson, 2016) studies have reported that individuals usually manage to adjust their energy intake (EI) when they perform sustained physical activity. Complete energy compensation is usually not observed at a single meal following an acute exercise session (Schubert, Desbrow, Sabapathy, & Leveritt, 2013), but at best a partial compensation can be observed over the following hours, days or weeks (Charlot & Chapelot, 2013; King, Tremblay, & Blundell, 1997; Pomerleau, Imbeault, Parker, & Doucet, 2004; Whybrow et al., 2008) indicating that the increase in EI is very likely progressive, but evidence is lacking to describe its kinetics.

Multi‐day military training expeditions offer an interesting opportunity for studying energy compensation since they submit individuals to mandatory long and repeated periods of sustained physical activity, resulting in high energy expenditure (EE). Surprisingly, results are generally characterized by what some have called “voluntary underconsumption” (Shukitt‐Hale, Askew, & Lieberman, 1997). This phenomenon, which is characterized by inadequate EI during high EE, occurs even when the total energy content of available foods largely exceeds energy requirements (Askew et al., 1987; Cline, Tharion, Tulley, Hotson, & Lieberman, 2000; Edwards & Roberts, 1991; Edwards, Roberts, Mutter, & Moore, 1990; Hoyt et al., 1991, 1994; Jones, Jacobs, Morris, & Ducharme, 1993; King et al., 1992; Lester et al., 1993; Lichton, Miyamura, & Mcnutt, 1988; Morgan et al., 1988; Popper et al., 1987; Roberts, Mcguire, Engell, Salter, & Rose, 1989; Schoeller, Field, & Delany, 1988; Shukitt‐Hale et al., 1997; Thomas et al., 1995). When prolonged, the energy deficiency caused by this negative energy balance (EB) (Loucks, 2004) or low energy availability (EA) (Loucks, Kiens, & Wright, 2011) leads to the loss of body mass (BM), either fat or fat‐free mass (Tassone & Baker, 2017), which may potentially impair physical and cognitive performance, and reduce mood (Cherif, Roelands, Meeusen, & Chamari, 2016; Church, Gwin, Wolfe, Pasiakos, & Ferrando, 2019; Karl et al., 2015; Murphy, Carrigan, Philip Karl, Pasiakos, & Margolis, 2018).

One hypothesis for the inadequate EI and lack of consistent progressive compensation is a ceiling effect. Sometimes called the “alimentary limit,” this effect has been observed during multiday endurance events: participants were not able to increase their EI above 2.5 times their resting metabolic rate (RMR) (Thurber et al., 2019). Considering that a typical male soldier's RMR is ~7.5 MJ (~1,800 kcal), the "alimentary limit" would be expected to be ~18.8 MJ (~4,500 kcal), a much higher value than the mean daily EI (~11.8 MJ or ~2,800 kcal) that was reported in previous studies (Askew et al., 1987; Cline et al., 2000; Edwards & Roberts, 1991; Edwards et al., 1990; Hoyt et al., 1991, 1994; Jones et al., 1993; King et al., 1992; Lester et al., 1993; Lichton et al., 1988; Morgan et al., 1988; Popper et al., 1987; Roberts et al., 1989; Schoeller et al., 1988; Shukitt‐Hale et al., 1997; Thomas et al., 1995). Thus, the incomplete energy compensation of high levels of EE seems more likely related to certain characteristics of the expedition and training conditions: total energy content of the provided rations, their ease of preparation, palatability, acceptability, diversity, the water availability (King et al., 1992), the stress of completing the training expedition (Fairbrother et al., 1995; Jacobs et al., 1989), the nature and intensity of physical activity (Charlot & Chapelot, 2019; Hazell, Islam, Townsend, Schmale, & Copeland, 2016; Larsen et al., 2019), and the time allocated to prepare and eat the provided foods (Edwards et al., 1990; Fairbrother et al., 1995; Jones et al., 1990; Margolis, Rood, Champagne, Young, & Castellani, 2013; Popper et al., 1987). Thus, the challenge is to discriminate between the respective roles of environmental and physiological limitations and therefore to determine the feasibility of appropriate strategies, so that individuals spontaneously adjust their EI when faced with sustained high levels of EE.

In a previous study (Charlot, Chapelot, Colin, & Bourrilhon, 2020), we reported that French soldiers potently increased their EI during the second half of a 14‐day expedition in Greenland. Energy equilibrium was even reached, and no significant BM change was observed at the end of the expedition. In this study, most of the limitations impeding adequate food intake were alleviated (rations composed of a large selection of easy‐to‐use, highly palatable and familiar foods and sufficient time allocated to prepare and eat the food). Nevertheless, the instability of the weather led participants to make several‐day halts and improvise their route and this may have influenced outcomes. Thus, this observation of efficient energy compensation during a high EE military expedition requires confirmation. In the same study (Charlot et al., 2020), some components of hedonic eating behavior (Berridge, 1996; Berridge, Robinson, & Aldridge, 2009) were assessed and "explicit liking" (EL) of high‐fat and/or sweet foods were correlated with changes in anthropometry. This potential modulation of food reward deserves to be explored more thoroughly.

The objective of the present study was therefore to confirm and then to analyze the characteristics of energy compensation, during a 15‐day expedition in the cold, with conditions designed to facilitate spontaneous food intake. We hypothesized that (a) a progressive increase of EI leading to a neutral EB and/or to sufficient EA (>30 kcal or 125 kJ·kg of body fat‐free mass [BFFM]^−1^·day^−1^) would occur and induce no or at worse slight BM changes, (b) eating behavior components such as hunger scores and reward value of food, would be closely related to EI and provide information about energy homeostasis under such harsh conditions.

## 2 MATERIALS AND METHODS

### 2.1 Design

**Figure 1:**
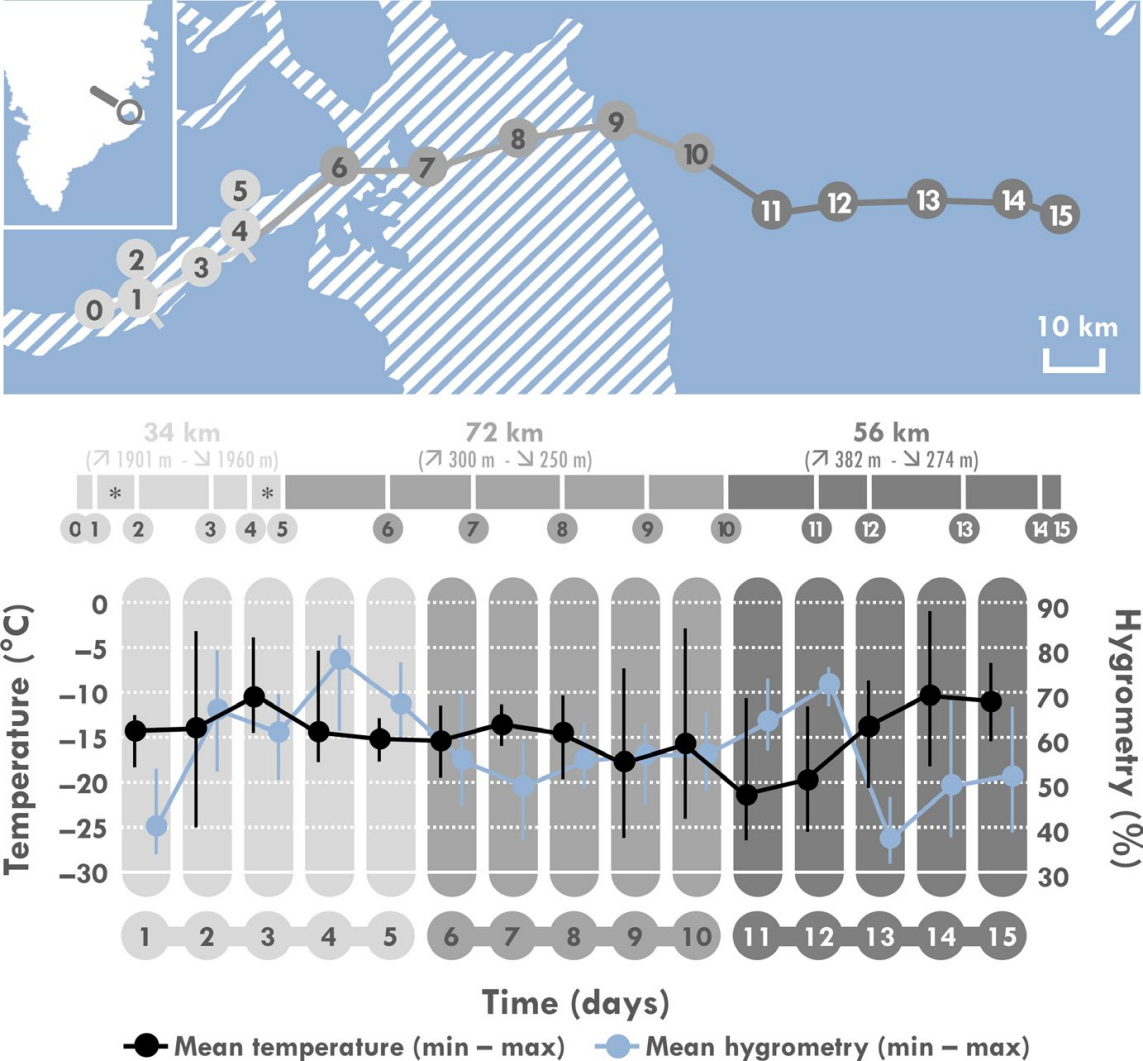
Details of the route of the expedition and meteorological conditions. *Participants realized a loop in cross‐country skis during these days

French soldiers planned this expedition in Greenland as part of an extreme cold inoculation program. They travelled from West to East on the Liverpool Land in Greenland (Figure 1). Participants skied most of the time while towing a pulka (from ~60 kg at the start to ~40 kg at the end of the expedition) except on D2 and D5 during which they cross‐country skied without pulka. Although they brought food for 21 days, the expedition lasted only 15 days, due to a quicker than expected pace. They were given a paper notebook on which they were requested to (a) evaluate their appetite, thirst, and fatigue feelings before breakfast and before dinner, (b) report all foods and beverages consumed over the whole 24 hr, and (c) assess the perceived reward value of foods prior to dinner once every 5 days. Each participant continuously wore a wrist heart rate (HR) monitor to estimate EE. Finally, BM and body fat‐mass were measured before breakfast on the morning before D1 and the day following their arrival (D16).

### 2.2 Participants

The participants were 12 male soldiers specialized in mountain environment activities (skiing, climbing, and snowshoeing) and familiarized with extreme conditions (cold and altitude). All were briefed several months before leaving France. This study was performed at the request of the Armée de Terre and approved by the scientific leadership of the French Armed Forces Biomedical Research Institute. This study required no invasive measurements and did not impose unfamiliar tasks to the participants. In this case, we were exempted according to the Institute regulation to obtain an ethical approval from a civilian Committee as long as the experiment was realized in accordance with the Declaration of Helsinki. They were therefore informed of the benefits and risks of the investigation prior to giving their written consent. All participants were screened by a military physician and had to be healthy. Their mean main characteristics are shown in Table 1.

**Table 1:** Participant's characteristics

Measurements	Unit	Value
Age	year	32.2 ± 5.8 [22–42]
Height	cm	178 ± 6 [170–188]
Body mass	kg	73.8 ± 6.1 [64.2–82.8]
Body mass index	kg/m	23.4 ± 1.5 [20.7–25.6]
Body fat mass	% of body mass	10.1 ± 2.4 [5.9–14.7]
Calculated resting metabolic rate	MJ/d	7.3 ± 0.4 [6.8–7.9]
Cooper performance	m	3,279 ± 110 [3100–3450]
Estimated *V*O_2max_	ml·min^−1^·kg^−1^	61.9 ± 2.5 [58.0–65.8]
Weekly physical activity	hr	9.1 ± 3.6 [4–15]

Note

Data are represented as means ± standard deviation [minimal value−maximal value].

Abbreviation: *V*O_2max_, maximal oxygen uptake.

### 2.3 Measurements

#### 2.3.1 Estimation of maximal oxygen uptake

Maximal oxygen uptake (*V*O_2max_) was estimated using their last Cooper 12‐min run test performance (Cooper, 1968) realized between 3 and 5 months before the beginning of the expedition. The aim of this traditional military test is to run as far as possible within 12 min. It has been shown to display a correlation coefficient between 0.897 and 0.920 with actual values (Cooper, 1968; Grant, Corbett, Amjad, Wilson, & Aitchison, 1995).

#### 2.3.2 BM and composition

On the morning before leaving for Greenland (D‐1), the participants had their BM and body composition assessed at a hotel in Reykjavik (Iceland). The measurements were performed before breakfast, with an empty bladder and using a calibrated bioimpedance meter scale (Tanita BC545N), with participants wearing only their underwear.

#### 2.3.3 Energy intake

Before the expedition, each participant packed 21 bags providing approximately 20 MJ (4,700 kcal) per day (see Appendix A for description). Seven different menus (Table 2) were conceived so that participants consumed each one only thrice during the expedition. To facilitate EI, these menus consisted of familiar and appreciated foods. To this purpose, a list of usual foods contained in the lyophilized French rations and foods that they usually bought for expedition in the cold or in altitude was established. Months before the study, a sample of soldiers (*n* = 5) was asked to remove from the list all food items that they estimated inappropriate for the Greenland expedition regarding their taste or their difficulties to prepare or to eat. Moreover 4 months before the expedition, all the participants were individually asked whether they wanted to replace food items they did not or weakly appreciate. Finally, 2 months later, they performed a three‐day training in the French Alps to test the material and three of the planned menus were provided. Again, they were authorized to replace some food items. On each morning and evening during the expedition, participants were granted one full hour to melt snow and rehydrate the food items in their tent and eat their meal. In‐between, they had ready‐to‐eat food items in their inner pockets while skiing. The expedition chief scheduled breaks approximately each hour and allowed time to drink and eat resulting in 5 to 8 eating occasions by day. Participants were also allowed to eat unconsumed food from the prior menu or give foods to other participants. This aimed to mimic an ad libitum condition and avoid any factor limiting EI owing to potential unavailability of a specific desired food. It is to note that the expedition‐induced burden was very low with the only aim being traveling a fixed distance to reach the pick‐up area. Participants had no operational tasks and had the possibility to sleep comfortably more than 7 hr each night.

**Table 2:** Daily menu characteristics

# Menu	Mass	AdWat	EC	CHO	FAT	PRO	CHO	FAT	PRO
	(g)	(ml)	(MJ)	(g)	(g)	(g)	(% EC)	(% EC)	(% EC)
A	1,080	1,485	195	531	168	232	45.8	32.3	19.8
B	1,109	1,639	19.5	556	152	241	47.8	29.4	20.7
C	1,067	1,254	19.4	514	171	231	44.2	33.2	19.9
D	1,147	1,660	20.7	582	179	241	46.9	32.4	19.4
E	1,113	1,635	19.9	564	166	236	47.4	31.4	19.8
F	1,092	1,335	19.4	523	171	231	45.2	33.3	19.9
G	1,082	1,210	19.5	557	157	236	47.9	30.3	20.3
Mean	1,099	1,460	19.7	547	166	235	46.4	31.8	20.0
*SD*	27	193	0.5	25	9	5	1.4	1.5	0.4
CV	2.4	13.2	2.5	4.5	5.5	1.9	3.1	4.6	2.0

Abbreviations: AdWat, volume of water to add; CHO, carbohydrate; CV, coefficient of variation; EC, energy content; PRO, protein.

The order of these menus was randomized to preclude that on any particular day all participants ate the same one. Before leaving camp and at the end of each day, participants had to complete a form on which they were asked to accurately report which items they had consumed, the amount eaten (in % of the portion) and the time of their consumption (in hours). These assessments were realized in real time for the meals consumed inside the tents (breakfast and dinner). For the foods consumed during the day, their recollection was facilitated by the fact they conserved all wrappings of the fully and partially unconsumed items. Based on these data, daily energy and macronutrient intake were calculated for each participant using the nutritional composition tables provided by the food manufacturers.

Each morning, participants prepared two 1‐L thermos bottles of drinks by melting snow (water, tea, infusion, coffee…). They had the possibility to repeat this process in the evening if they desired. Participants knew the volume of the bottles and the cups they drank from, facilitating the estimation of the consumed volume.

Two weeks before the beginning of the expedition, participants completed a 3‐day food diary during a representative work week (from Tuesday to Thursday) allowing the calculation of usual EI.

#### 2.3.4 Energy expenditure

Participants were outfitted with a Fenix 5X Saphhire watch (Garmin) equipped with a wrist‐worn optical HR monitor continuously recording at the frequency of one measurement every minute, except for a 3‐hr recharging period scheduled between D8 and D10 according to the participants. A week prior to the expedition, participants had a period of familiarization with the watch, notably for learning how to wear it, that is, 2 cm above the wrist bone and snug without compressing vessels as stated by the manufacturer.

The participants’ HR data were converted in HR reserve (HRR), that is, maximal minus resting HR, using the nocturnal resting HR and the theoretical maximum (210 − 0.66 × age) (Bruce, Fisher, Cooper, & Gey, 1974). Hiilloskorpi and colleagues (Hiilloskorpi, Pasanen, Fogelholm, Laukkanen, & Manttari, 2003) have proposed two equations for predicting EE with BM and HRR at low (1–3 METS) and high (>3 METS) intensities:

(1) $$\text{EE at low intensities}\;\left(\text{kcal}\cdot \min^{‐1}\right)=\left(0.449+0.0627\times \text{HRR}+0.00743\times \text{BM}+0.00100\times \text{HRR}\times \text{BM}\right).$$

(2) $$\text{EE at high intensities}\;\left(\text{kcal}\cdot \min^{‐1}\right)=\left(1.044+0.0250\times \text{HRR}+0.01088\times \text{BM}+0.00177\times \text{HRR}\times \text{BM}\right).$$

In their subjects' sample, maximal HR at low intensities was 130 bpm. Thus, we used equation 1 for HR under 130 bpm and equation 2 for HR ≥130 bpm. For each minute of the whole day calculated EE was cumulatively added and converted in MJ/day.

#### 2.3.5 Energy balance and availability

The EB (MJ/day) was calculated by subtracting the EE from EI (both in MJ/day), negative values indicating energy deficiency. EA (kJ·kg BFFM^−1^·day^−1^) was calculated using the following formula: (EI − Ex‐EE)/BFFM where EI and Ex‐EE are expressed in kJ/day and BFFM in kg. Daily EE corresponds to the sum of the RMR, dietary induced thermogenesis (DIT), and Ex‐EE. The RMR was calculated using predictive equations (Roza & Shizgal, 1984) and DIT was assumed to correspond to 10% of the RMR. Thus, Ex‐EE could be deduced by subtracting the RMR and DIT from the EE. This calculation has already been used during military training (Mullie, Clarys, De Bry, & Geeraerts, 2019). EA “quantifies the amounts of energy available for bodily functions after the energetic cost of exercise has been removed” (Loucks, 2004) and alterations of physiological processes have been observed under certain thresholds (Logue et al., 2020; Loucks et al., 2011). An energy replete state has been defined as 188 kJ or 45 kcal·kg BFFM^−1^·day^−1^ but it is generally recognized that low EA is reached below 125 kJ or 30 kcal·kg BFFM^−1^·day^−1^ (Holtzman & Ackerman, 2019; Logue et al., 2020; Loucks et al., 2011).

#### 2.3.6 Subjective ratings

Sensations of hunger, thirst, and fatigue were assessed using visual analog scales (VAS). The participant had to draw a vertical dash on a horizontal 10‐cm scale preceded by the question: ‘‘How hungry/thirsty/fatigued do you feel?’’ and anchored at the left and right ends by “not at all” and “extremely,” respectively. The distance from the extreme left to the participant's vertical dash represented the rating score, expressed in mm. These scales have been shown to have a good level of reliability and reproducibility, and a certain power of predictability (Stubbs et al., 2000). Moreover each evening prior to dinner, ratings of perceived exertion (RPE) were assessed using a graduated 0 to 10 scale for physical activities practiced during the current day (Foster et al., 2001).

#### 2.3.7 Reward value of food

Finlayson and colleagues (Finlayson, King, & Blundell, 2007, 2008) developed the Leeds Food Preference Questionnaire (LFPQ) for measuring several components of food reward, that is, EL, implicit wanting and food preference (Dalton & Finlayson, 2014). We recently conceived and validated a shorter version of this questionnaire, called the Food Preference Questionnaire (FPQ‐S_16_), using only 16 food items and adapted for the French population (Charlot, Malgoyre, & Bourrilhon, 2018). This version is less time‐consuming and appeared reasonably reliable, with correlation coefficients with the LFPQ between 0.83 and 0.88. One limitation is that this version only assesses EL and food preference but not implicit wanting since reaction time is not measured (questionnaire performed on paper, see below). The choice and presentation of foods of the FPQ‐S_16_ was found to be consistent with the recent recommendations provided by the team that developed the LFPQ in order to adapt it to other cultures (Oustric et al., 2020). Given the difficulties of using a computer during such expeditions, notably due to the weight of the equipment and the deleterious consequences of very low temperatures on computers, the FPQ‐S_16_ was adapted to paper. This procedure was already successfully used in our previous study (Charlot et al., 2020). Briefly, the questionnaire consists in 16 pictures of food items categorized according to their fat content (high‐fat [HF] or low‐fat [LF]) and taste (sweet [SW] or savory [SA]). This results in four specific food categories (HF, LF, SW, and SA) with eight food items in each category and four combined categories (HFSW, HFSA, LFSW, and LFSA) with four food items in each one. To assess EL, participants had first to answer to the question “How pleasant would it be to taste some of this food now?” for each food on a VAS. Then, to assess food preferences, the 16 food items were presented on the same page in a randomized order. Participants were then asked to rank these items in four qualitatively rated groups, ranging from the group that contained the four food items that they "wanted to eat the most now" (++), to the group that contained the four food items that they "wanted to eat the least now" (−−). The number of food items from a specific (HF, LF, SW, or SA) or combined (HFSW, HFSA, LFSW, or LFSA) category was multiplied by the group coefficient (3 for ++, 2 for +, 1 for ‐, and 0 for −−) and then scores from each group added. The score ranges for the specific (8 food items) and combined (4 food items) categories were therefore 4–20 and 0–12, respectively. Absolute scores were used to assess EL and appeal bias scores were calculated for food preference by subtracting a score from each category with its opposite one (e.g., HF vs. LF or HFSA vs. LFSA). Thus, a positive or negative score indicated a preference for one category over the other. This procedure was conducted prior to dinner in a hungry state once every 5 days (D5, D10, and D15).

### 2.4 Statistical analyses

Given the highly variable conditions of such expeditions, for example, weather and distance travelled per day (see Results for details), we divided the temporal analysis into three periods and values were averaged on a 5‐day basis: D1–5, D6–10, and D11–15. Comparing day‐to‐day differences would have required highly conservative corrections for multiple comparisons and would have been subjected to artifacts due to certain single‐day events. EI was calculated in absolute and in relative values, that is, percentage of total available energy consumed (TAEC). All variables were checked for normality using a Shapiro‐Wilk test followed by skewness and kurtosis. Paired Student's *t*‐test was used for BM and composition changes. Most variables were compared using a mixed‐model repeated‐measures 3 × 1 ANOVA with period (D1–5, D6–10, and D11–15) as the within‐subject factor. To explore the kinetics of the expected energy compensation during the day, an independent analysis of four 4‐hr within‐day time‐periods (06–10, 10–14, 14–18, and 18–22 hr) was proceeded using a mixed‐model repeated‐measures 3 × 4 ANOVA with periods of the expedition (D1–5, D6–10, and D11–15) and the day (06–10, 10–14, 14–18 and 18–22 hr) as the within‐subject factors. To determine the status of EB (negative, neutral, or positive), a mixed‐model repeated‐measures 3 × 2 ANOVA was proceeded with period (D1–5, D6–10, and D11–15) as the within‐subject factor and outcome (EE and EI) as the between‐subject factor. When the sphericity assumption was violated (Mauchly's test), a Greenhouse‐Geisser correction was used. The *post hoc* analyses were performed using Bonferroni's tests. Data are presented as the means ± *SD*. Significance was defined as *p < *.05. Analyses were performed using Jamovi software (1.2.9 version, the Jamovi project; retrieved from https://www.jamovi.org).

## 3 RESULTS

### 3.1 Details of the expedition

The details of the route, temperatures, and hygrometry are illustrated in Figure 1. Participants remained in the same locations for a total of 2 days (D2 and D5) to realize a loop in cross‐country skis. Mean temperature over the whole expedition was −14°C (min = −26 to max = −1°C), respectively. Weather was mostly sunny and wind speed was <30 km/hr. Total travelled distance was 162 km and mean daily distance was 10.8 ± 4.8 km (+163 ± 305 m and −166 ± 306 m). BM slightly but significantly decreased from 73.8 ± 6.1 to 73.0 ± 5.7 kg ([−3.5 to +0.3 kg]; *p = *.021) during the expedition, representing a mean relative modification of −1.13% [−4.4 to +0.4%]. Body fat mass percentage decreased from 10.1 ± 2.4 to 8.0 ± 1.8% ([−4.8 to −0.1%]; *p < *.001), representing a mean relative modification of −19.5% [−41.4 to −1.6%]. Of note, measurements performed 4 months earlier, during inclusion visits, showed that BM was relatively stable during this pre‐expedition period (+0.46 ± 0.96%).

### 3.2 Energy and macronutrient intakes

**Figure 2:**
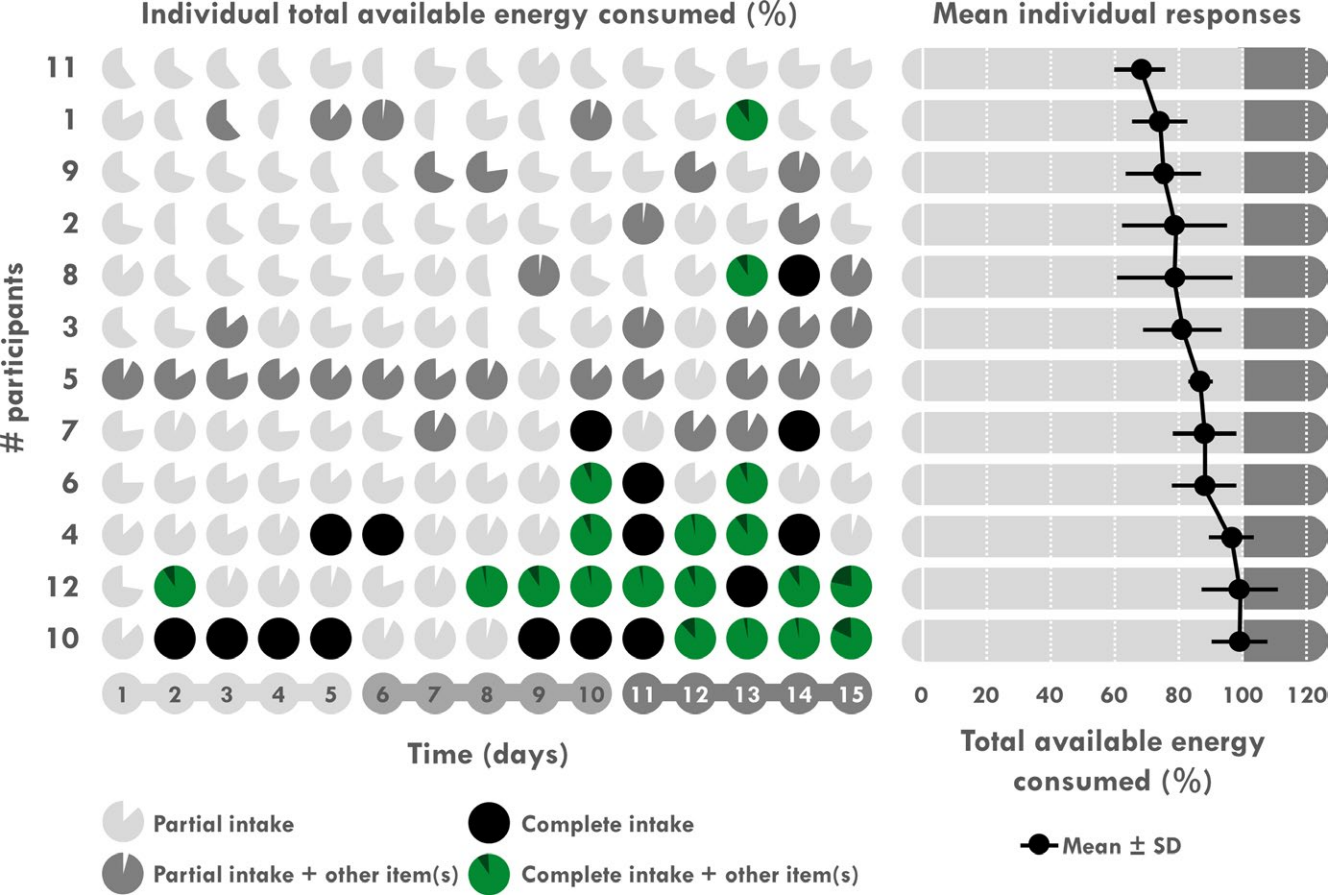
Individual daily energy intake relative to total energy content and mean individual responses

Over the expedition period, mean EI, EE, EB, and EA were 16.3 ± 2.0 MJ/day, 17.5 ± 1.7 MJ/day, −1.2 ± 2.4 MJ/day, and 102 ± 66 kJ/kg BFFM, respectively. EB was considered neutral since EE was not different from EI (*p = *.123). Mean EI was 2.31 ± 0.26 times their RMR (1.81–2.75). Mean percentage of TAEC was 84.0 ± 10.2% with a large individual variability ranging from 69% to 99% (Figure 2). Mean carbohydrate, fat and protein intakes were 458 ± 63 (46.6 ± 2.3% of EI), 157 ± 20 (36.3 ± 1.6%), and 166 ± 18 g/day (17.1 ± 0.8%), respectively. Mean water intake was 2.2 ± 0.6 L/day.

**Figure 3:**
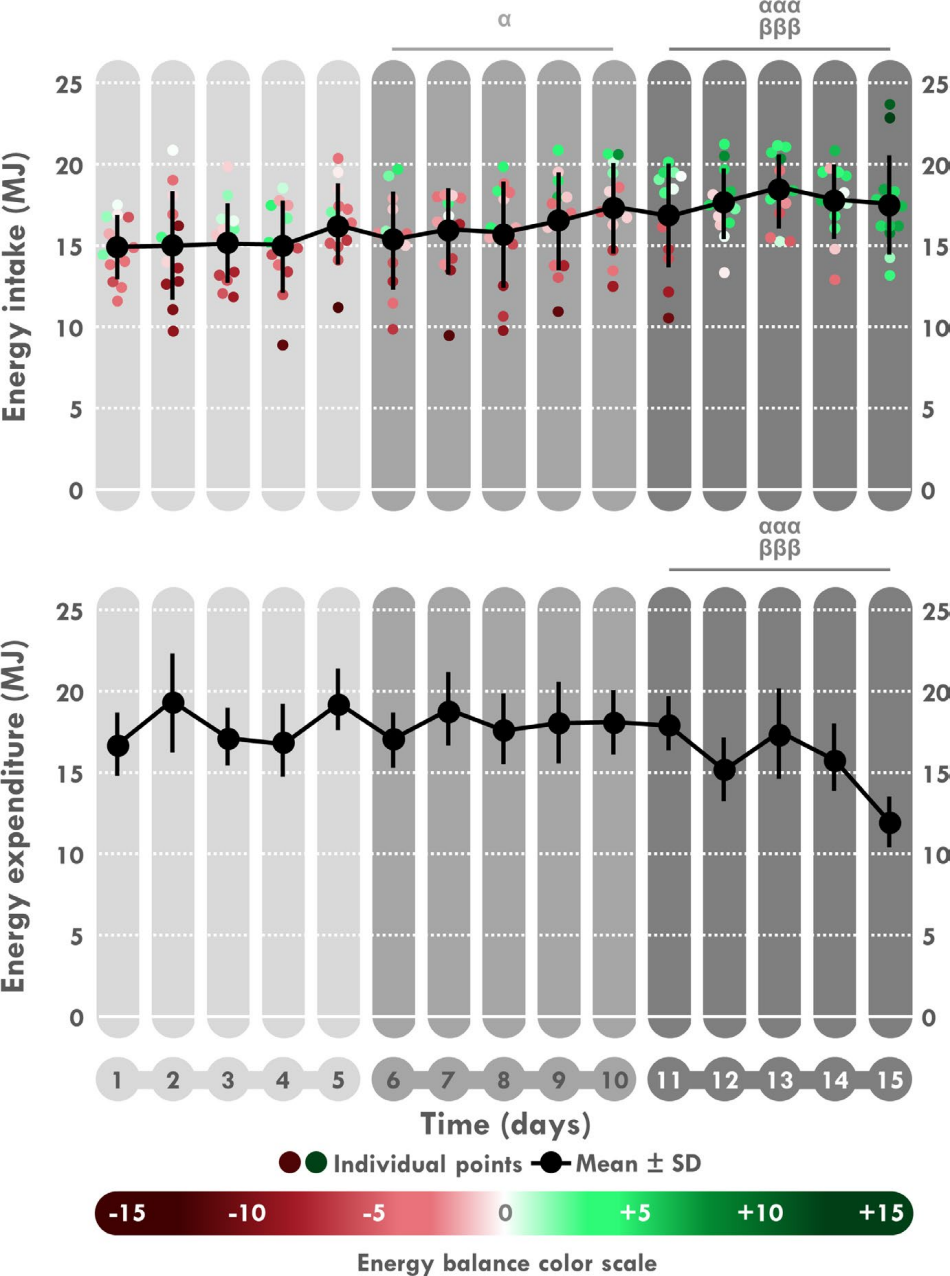
Mean and individual daily energy intake and expenditure. ^α^Different from D1 to 5, ^β^different from D6 to 10. One sign: *p < *.05, three signs: *p < *.001

**Figure 4:**
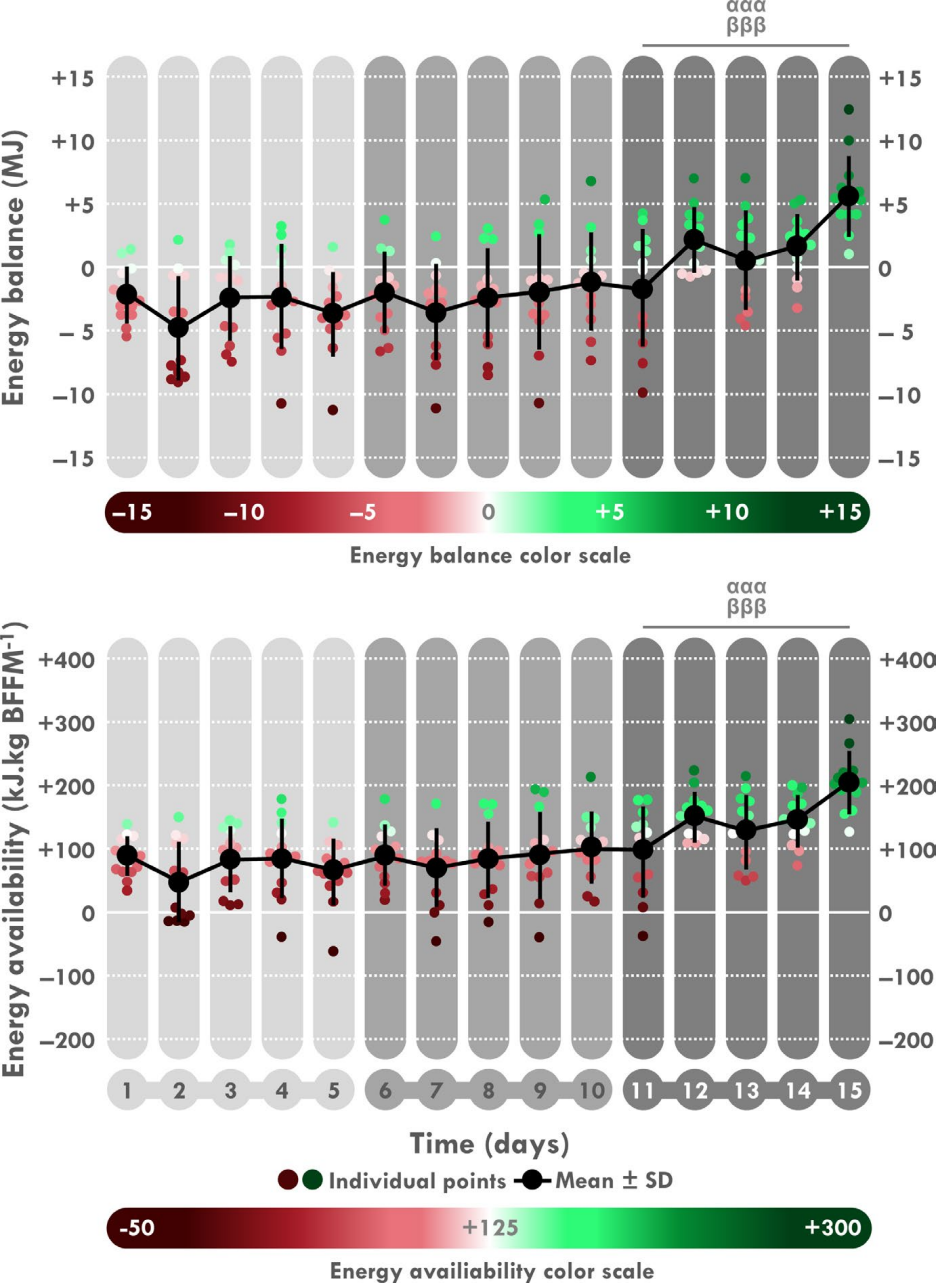
Mean and individual daily energy balance and availability. ^α^Different from D1 to 5, ^β^different from D6 to 10. Three signs: *p < *.001

**Figure 5:**
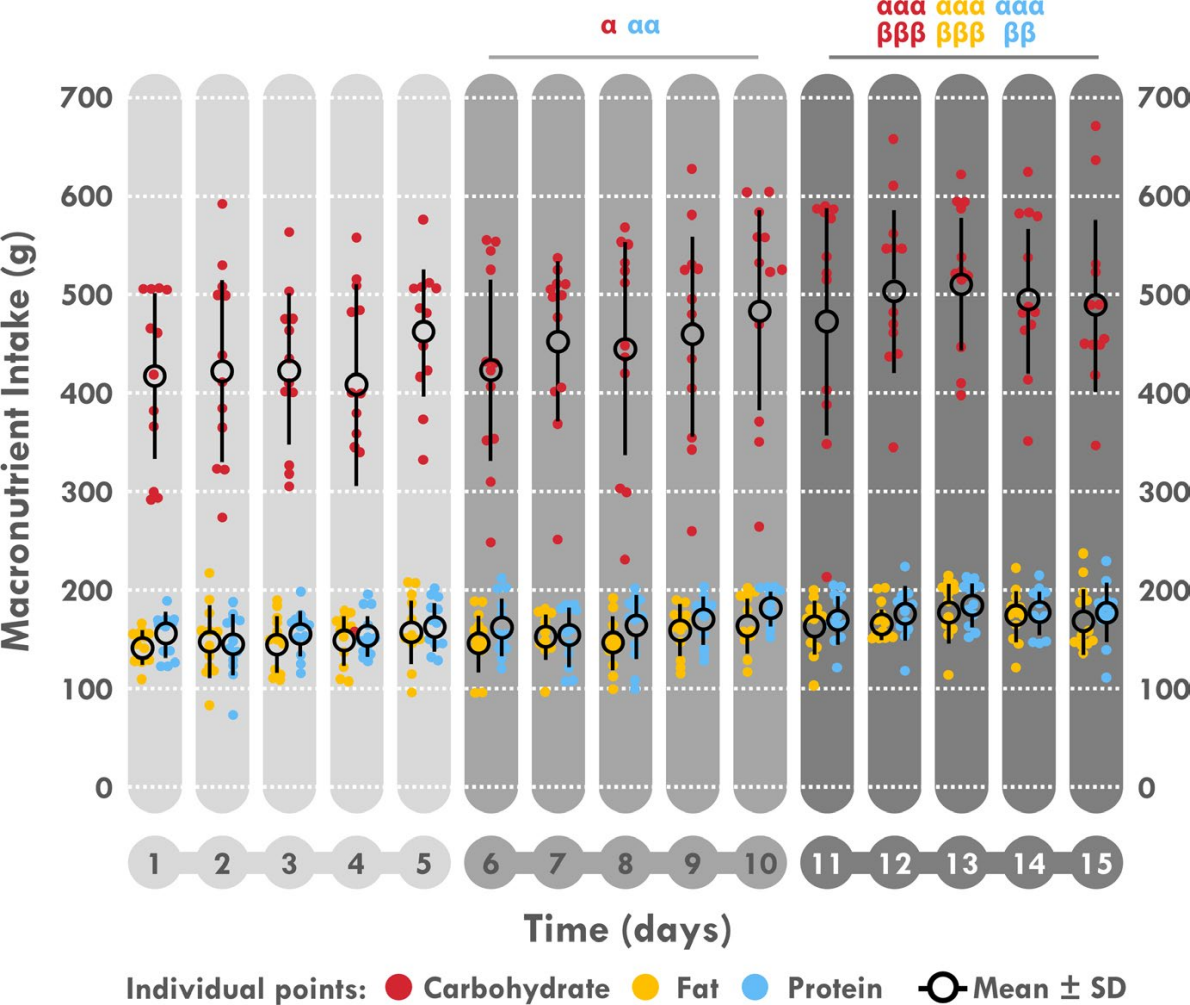
Mean and individual daily carbohydrate, fat and protein intake. ^α^Different from D1 to 5, ^β^different from D6 to 10. One sign: *p < *.05, two signs: *p < *.01, three signs: *p < *.001

When analyzed by 5‐day periods, a time effect was found for EI, EE, EB, and EA (*p < *.001 for all). As illustrated in Table 3 and Figure 3, a significant increase in EI was found during D6–10 compared to D1–5 (*p = *.012) and was still enhanced during D11–15 compared to D1–5 and D6–10 (*p < *.001 for both). Conversely, EE decreased during D11–15 compared to D1–5 and to D6–10 (*p < *.001 for all). Logically, EB and EA increased during D11–15 compared to D1–5 and to D6–10 (*p < *.001, Figure 4). EB was negative during D1–5 (EE > EI; *p = *.018) and neutral during the last two periods. Consistently, a time‐effect was found for TAEC which increases during D6–10 compared to D1–5 (*p* < .05) and during D11–15 compared to D6–10 and D1–5 (both *p < *.001). A time‐effect was also found for macronutrient intake in absolute values (g) but not in percentages of EI. Comparisons showed a significant increase in amount of carbohydrate, fat and protein intakes (Table 3 and Figure 5) during D11–15 compared D1–5 (*p < *.001 for all) and D6–10 (*p < *.001, *p < *.001 and *p = *.002, respectively) whereas this increase was only significant for carbohydrate and protein during D6–10 compared to D1–5 (*p = *.012 and *p = *.008, respectively). A time‐effect was found for water intake with a significant increase during D11–15 compared to D1–5 (*p = *.022).

**Table 3:** Mean energy expenditure and intake and subjective ratings for each period of the expedition

Measurement	Unit	D1–D5	D6–D10	D11–D15
Energy expenditure	MJ/day	18.3 ± 1.9	18.3 ± 2.0	15.9 ± 1.6^ααα, βββ^
Energy intake	MJ/day	15.3 ± 2.1*	16.1 ± 2.1^α^	17.6 ± 2.0^ααα, βββ^
Energy intake	% of TEC	78.5 ± 11.0	82.9 ± 10.8^α^	90.5 ± 10.0^ααα, βββ^
Energy balance	MJ/day	−3.0 ± 2.7	−2.2 ± 2.8	+1.7 ± 2.4^ααα, βββ^
Energy availability	kJ/kg BFFM	74 ± 41	88 ± 42	146 ± 34^ααα, βββ^
Carbohydrate intake	g/day	425 ± 66	452 ± 69^α^	495 ± 64^ααα, βββ^
Fat intake	g/day	148 ± 22	154 ± 20	169 ± 18^ααα, βββ^
Protein intake	g/day	154 ± 19	166 ± 20^αα^	177 ± 19^ααα, ββ^
Carbohydrate intake	% of EI	46.4 ± 2.6	46.6 ± 2.6	46.9 ± 2.3
Fat intake	% of EI	36.6 ± 2.0	36.1 ± 2.2	36.3 ± 1.3
Protein intake	% of EI	17.0 ± 1.1	17.3 ± 0.8	16.9 ± 0.9
Water intake	L/day	2.0 ± 0.6	2.1 ± 0.5	2.4 ± 0.6^α^
EI (06:00–10:00)	% of EI	20.6 ± 5.2	22.5 ± 6.3	21.5 ± 5.8
EI (10:00–14:00)	% of EI	16.9 ± 4.3	20.0 ± 4.6	23.3 ± 6.4^ααα^
EI (14:00–18:00)	% of EI	24.2 ± 7.6	18.7 ± 4.0	20.1 ± 6.6
EI (18:00–22:00)	% of EI	38.4 ± 10.8	38.8 ± 8.1	35.2 ± 9.8
Morning hunger score	mm (/100)	50 ± 19	56 ± 17	62 ± 17^ααα, β^
Evening hunger score	mm (/100)	62 ± 17	73 ± 17^αα^	73 ± 17^αα^
Morning thirst score	mm (/100)	56 ± 20	59 ± 20	64 ± 20^αα^
Evening thirst score	mm (/100)	64 ± 14	66 ± 17	68 ± 15
Morning fatigue score	mm (/100)	28 ± 9	31 ± 10	28 ± 9
Evening fatigue score	mm (/100)	42 ± 10^βββ^	53 ± 13	38 ± 17^βββ^
RPE	(/10)	53 ± 6^ββ^	62 ± 8	47 ± 12^βββ^

Note

Means ± standard deviation.

Abbreviations: EI, energy intake; RPE, rates of perceived exertion.

^α^Different from D1 to 5, ^β^different from D6 to 10, *EE different from EI. One sign: *p < *.05, two signs: *p < *.01, three signs: *p < *.001.

**Figure 6:**
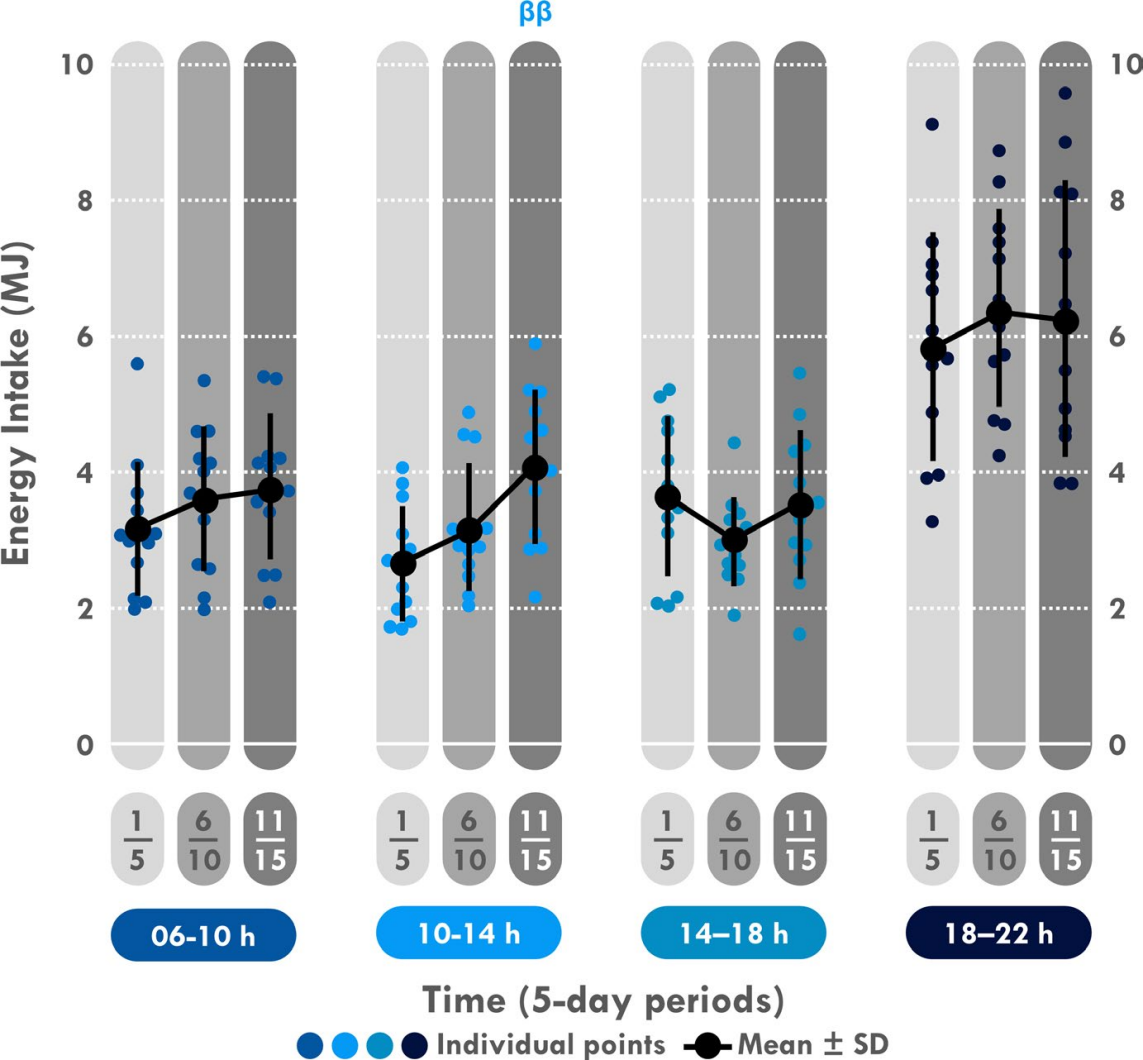
Mean and individual energy intake divided in four periods. ^ββ^Different from D6 to 10, *p < *.01

Concerning the analysis by 4 hr‐periods in absolute (Figure 6) and relative (Table 3) values, an interaction between periods of the expedition and periods of the day was found for both (*p = *.049 and 0.042, respectively). Post hoc tests revealed that EI only increased between 10:00 and 14:00 during D11–15 compared to D1–5 (*p = *.002 and *p* < .001, respectively).

Correlations showed that the expedition‐induced modifications in BM (in %) was positively associated with mean EI (*r = *0.598, *p = *.040) and mean carbohydrate intake (*r = *0.622, *p = *.031). No other correlation reached statistical significance.

### 3.3 Subjective scores and food reward components

**Figure 7:**
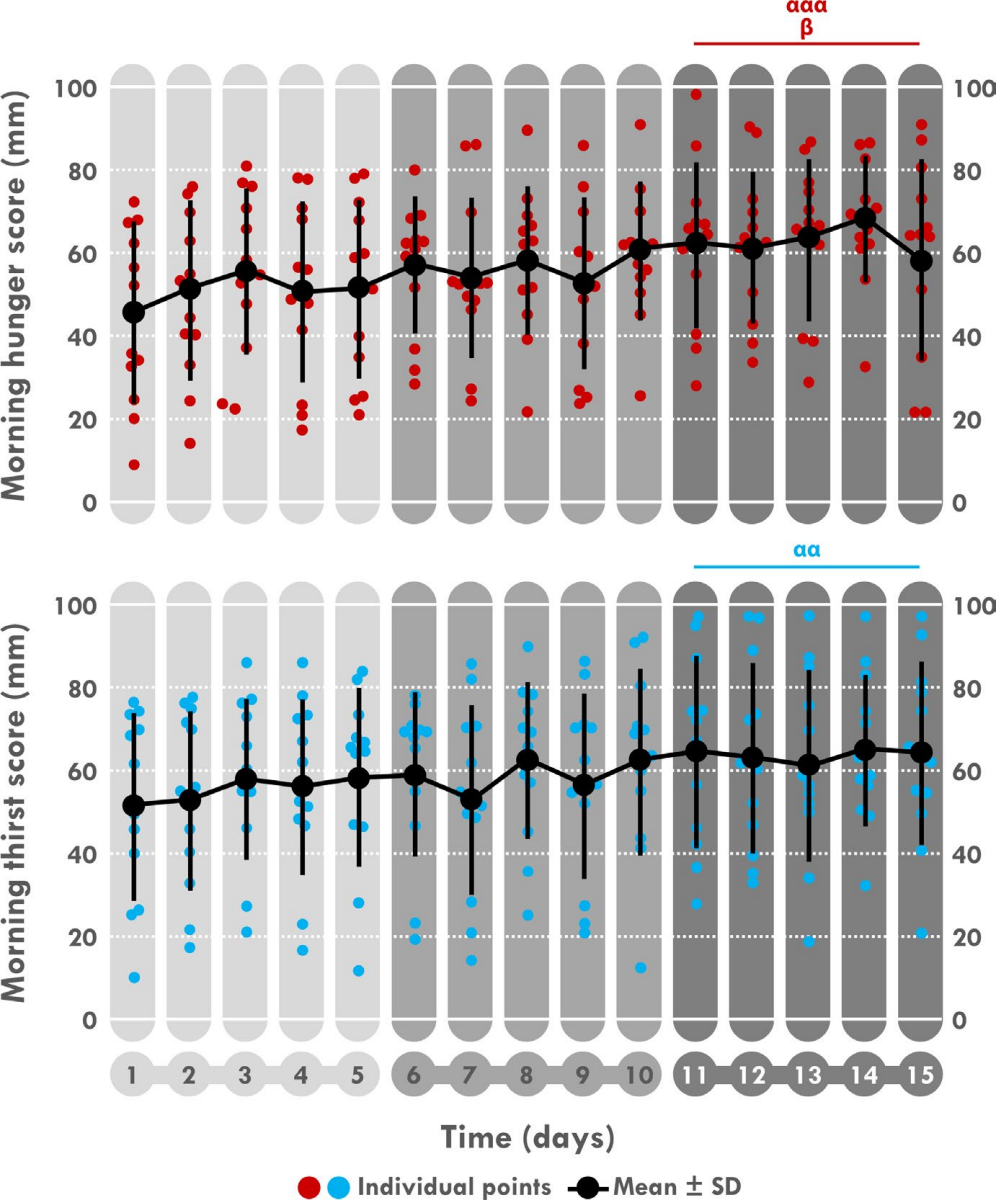
Mean and individual daily morning hunger and thirst scores. ^α^Different from D1 to 5, ^β^different from D6 to 10. One sign: *p < *.05, two signs: *p < *.01, three signs: *p < *.001

A time effect was found for the morning and evening hunger scores (*p < *.001 and *p = *.003, respectively) and for the morning thirst scores (*p = *.011), and the evening fatigue (*p < *.001) and RPE scores (*p < *.001). Morning hunger scores increased during D11–15 compared to D6–10 (*p = *.050) and to D1–5 (*p < *.001) (Table 3; Figure 7) and evening hunger scores increased during D6–10 and D11–15 compared to D1–5 (*p = *.003 for both). Morning thirst score increased during D11–15 compared to D1–5 (*p = *.009). Evening fatigue score and RPE increased during D6–10 compared to D1–5 (*p = *.001 and *p = *.005, respectively) and D11–15 (*p < *.001 for both).

**Figure 8:**
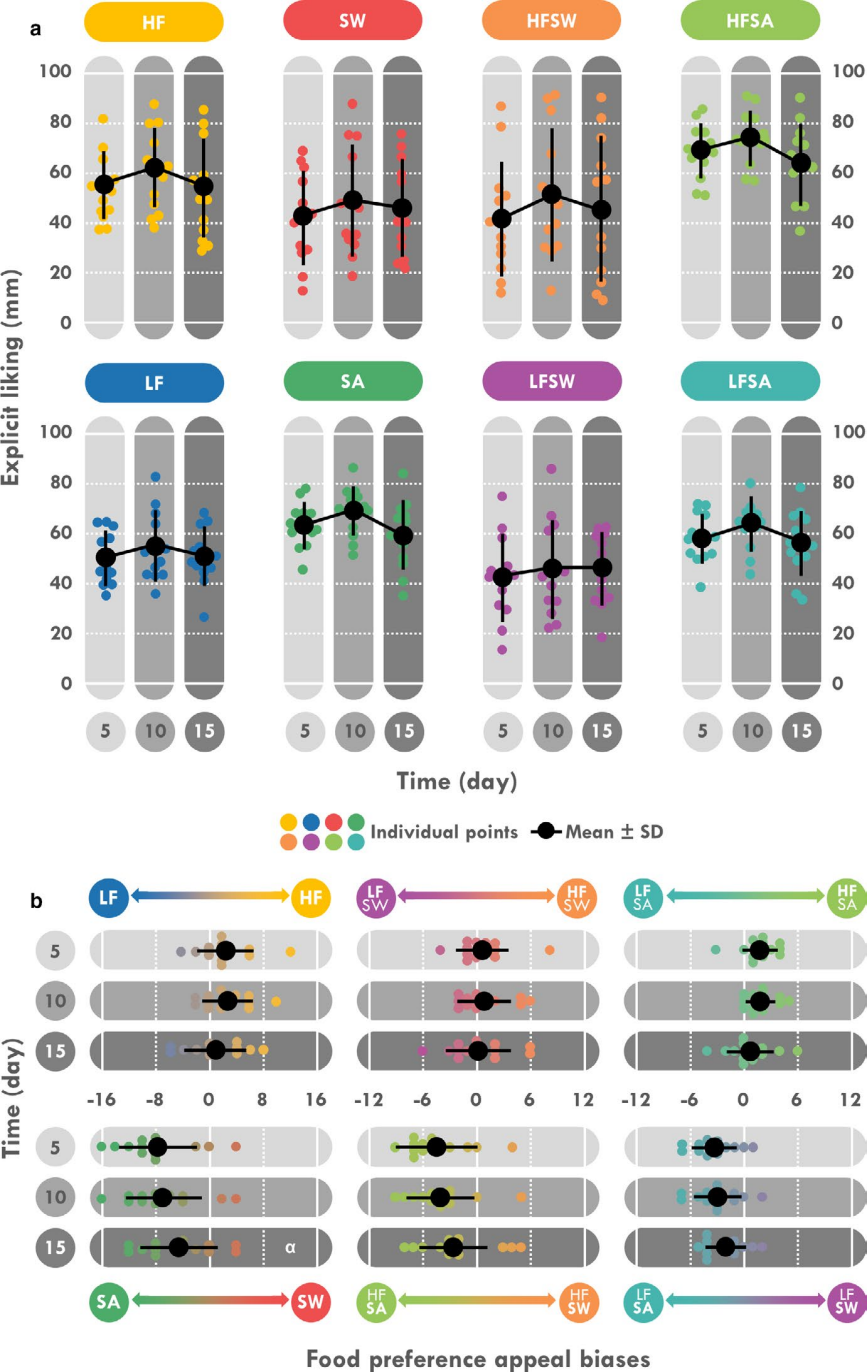
Mean and individual explicit liking scores (a) and food preference appeal biases (b). HF, high‐fat; LF, low‐fat; SW, sweet; SA, savory. ^α^Different from D1 to 5, *p < *.05

No time effect was found for variables of EL (Figure 8a) and only the sweet versus savory bias score (*p = *.046) for food preference (Figure 8b). This bias increased during D11–15 compared to D1–5 (*p = *.026) reflecting a higher preference for sweet foods. No significant correlation was found between any subjective scores or food reward results with any component of intake, expenditure or BM.

## 4 DISCUSSION

The aim of this study was to confirm the complete energy compensation that we had previously observed in soldiers participating in a 14‐day expedition in Greenland (Charlot et al., 2020) and analyze its characteristics during a similar 15‐day expedition in the cold. This former result was noticeable since compensation is usually reported to be weak and delayed. It was hypothesized that the practical adaptations in food supply and eating conditions reduced the previously described "voluntary underconsumption." The present study, based on a similar paradigm, actually confirmed this complete energy adjustment with an increased EI between the 5th and 10th days, leading to a positive EB and normal EA values between the 11th and 15th days. Although a statistically significant BM loss was observed, its amount (800 g) was trivial. Consistently, hunger and thirst progressively increased during the expedition, and although reward variables were mostly unaffected, a certain preference for sweet compared to fat foods was observed. These results indicate that energy homeostasis is a potent mechanism that may operate even in extreme environmental and physical conditions if appropriate food supply and eating conditions are proceeded.

Compared to previous reports of multiday expeditions, our results show an unusually high EI. Actually mean EI usually ranges from 8.2 to 15.2 MJ for a 10.3 to 20.6 MJ EE (Askew et al., 1987; Cline et al., 2000; Edwards & Roberts, 1991; Edwards et al., 1990; Hoyt et al., 1991, 1994; Jones et al., 1993; King et al., 1992; Lester et al., 1993; Lichton et al., 1988; Morgan et al., 1988; Popper et al., 1987; Roberts et al., 1989; Schoeller et al., 1988; Shukitt‐Hale et al., 1997; Thomas et al., 1995) whereas our participants consumed ~16.3 MJ for a mean ~17.5 MJ EE. Our nutritional conditions seem to have been optimal for an ad libitum food intake, allowing individuals to maintain energy homeostasis. Consistently, BM loss (−0.05 g/day) was in the low range of previous results (from −0.01 to −0.30 g/day) (Askew et al., 1987; Cline et al., 2000; Edwards & Roberts, 1991; Edwards et al., 1990; Hoyt et al., 1991, 1994; Jones et al., 1993; King et al., 1992; Lester et al., 1993; Lichton et al., 1988; Morgan et al., 1988; Popper et al., 1987; Roberts et al., 1989; Schoeller et al., 1988; Shukitt‐Hale et al., 1997; Thomas et al., 1995). Interestingly, the so‐called "alimentary limit" (2.5 fold the RMR (Thurber et al., 2019) was overtaken by one, four and six of our participants during the D1–5, D6–10 and D11–15 periods, respectively. This strongly suggests that this hypothetical limit is not mandatory and that even expeditions in harsh conditions with physical activities inducing a daily EE >2.5 fold RMR, do not preclude a spontaneous adequate energy compensation. Importantly, our participants were not forced nor even encouraged to eat by their commander. Therefore, EI may be considered as spontaneous. The fact that we found similar capacities of complete energy compensation and therefore energy homeostasis with two different samples of subjects, in a similar cold environment and with similar levels of physical activities (Charlot et al., 2020), supports the efficiency and the robustness of our results.

Providing available energy in excess compared to expected EE is not enough to ensure that EI will accurately compensate for EE. The number of internal and external moderators framing the eating context that determines food intake is large. Eating milieu or ambience (temperature, hygrometry, locations, time of eating, social interactions…) (Charlot, Faure, & Antoine‐Jonville, 2017; Hirsch, Matthew Kramer, & Meiselman, 2005; Stroebele & De Castro, 2004), food characteristics (palatability, variety, portion size, serving temperature…) (De Graaf, Kramer, et al., 2005; Kramer, Lesher, & Meiselman, 2001), not forgetting stress (Zellner et al., 2006; Zellner, Saito, & Gonzalez, 2007), are potential factors that may suppress or enhance EI. Military expeditions cumulate numerous and sometimes specific moderators that have been extensively described in a book called “Not Eating Enough” (Institute of Medicine, 1995). Identifying the moderators favoring intake is a main military objective (De Graaf, Cardello, et al., 2005; De Graaf, Kramer, et al., 2005; Hirsch et al., 2005; Kramer et al., 2001) to help limiting negative EB and its potential deleterious consequences. In this and our previous studies (Charlot et al., 2020), we attempted to reduce most of these negative moderators in managing both food characteristics and eating milieu. The food selection was rationalized to provide the most appropriate items regarding the cold arctic context, that is, a mix of lyophilized and ready‐to‐eat foods to consume according to the situation: in the tent with melted snow or skiing without preparation, respectively. Moreover the time pattern was designed (a) to allow enough time under the tents in the morning and in the evening for rehydrating lyophilized items and eating quietly, (b) to spread regular breaking time bouts during the diurnal nomad period with free‐time for consuming the available ready‐to‐eat food items. Finally, trading and giving away foods between participants was authorized to avoid a potential under‐eating effect due to the limited size of daily menus (fixed number of items by participant) and individual items (one portion). It has been previously reported that soldiers skipped eating in order to accomplish their missions during training courses (Institute of Medicine, 1995). It is likely that schedule constraints contribute to the lack of energy compensation. Here, we show that simple and practical solutions should be planned to help spontaneously increase EI.

The present study brought new details about the temporal modalities of this energy compensation. As in our previous expedition (Charlot et al., 2020), a progressive increase in EI occurred as soon as the second week (D5–10) with constant high level of EE, reaching neutral EB. This difference was still enhanced at the end of this week (D11–15), although EE slightly decreased, such that EB became positive at the end of the expedition (+1.7 ± 2.4 MJ) and EA exceeded the low EA threshold (125 kJ·kg BFFM^−1^·day^−1^). The decrease in EE in the last third was explained by the management of their progression. Indeed, their progression was faster than expected in the beginning of the expedition and they had to slow down in order to reach the destination at the right time.

A coupling between EI and EE has first been proposed in the 1950s by Mayer et al. (1956) who reported a J‐shaped curve between EI and the amount of professional physical activities in Indian mill workers. While EI seemed dysregulated (paradoxically more important than EE) in workers with sedentary tasks, it was then correlated linearly with the increase in EE. A systematical review by Beaulieu et al. (2016) based on 10 studies recently confirmed this relationship. Moreover there is a growing body of evidence linking the different components of EE (RMR and Ex‐EE) and EI (Blundell et al., 2020; Blundell, Gibbons, Caudwell, Finlayson, & Hopkins, 2015). Military studies showed discrepant results with an increase (Cline et al., 2000; Edwards & Roberts, 1991; Edwards et al., 1990; Jones et al., 1993), a stagnation (Askew et al., 1987; Thomas et al., 1995) or a decrease (Edwards & Roberts, 1991; Edwards et al., 1990; King et al., 1992; Lichton et al., 1988) in EI over 6 to 30 days. Moreover these temporal fluctuations were almost never statistically assessed. A series of studies from Stubbs and colleagues failed to observe a significant compensatory EI in men and women (Stubbs et al., 2004; Stubbs, Sepp, Hughes, Johnstone, Horgan, et al., 2002; Stubbs, Sepp, Hughes, Johnstone, King, et al., 2002) during a 7‐day laboratory stay with two daily different levels of exercise (moderate or high) and therefore high and moderate levels of EE (Stubbs, Sepp, Hughes, Johnstone, Horgan, et al., 2002; Stubbs, Sepp, Hughes, Johnstone, King, et al., 2002). On a longer term (16 days), they observed a weak and incomplete 30% energy compensation in the high but not in the moderate level condition, and in men but not in women, with a sustained negative EB (–4.7 MJ/day in men) (Whybrow et al., 2008). This led the authors to conclude that “it takes presumably a number of weeks for EI to match EE to achieve a new balance” (Blundell, Stubbs, Hughes, Whybrow, & King, 2003). Interestingly, EE in the high level condition of their male subjects was close to the one in the present study (16.7 versus 17.5 MJ/day). Since our participants succeeded in matching EI and EE and reach neutral EB in about only ten days, this suggests that environmental conditions (laboratory or expedition) may modulate the energy homeostasis process. Although the main determinants of this facilitating effect have to be assessed, some of them can be raised: cold weather (Charlot et al., 2017), food palatability (De Graaf, Kramer, et al., 2005), daily activities (Koball, Meers, Storfer‐Isser, Domoff, & Musher‐Eizenman, 2012), conviviality (de Castro & de Castro, 1989). Thus, our observations in extreme environment and physical activities may not be relevant to laboratory or everyday life conditions and may paradoxically optimize the process leading to energy compensation. In concordance with highly controlled laboratory studies (Charlot & Chapelot, 2019; Finlayson, Bryant, Blundell, & King, 2009), we observed a large interindividual variability in the level of EI and energy compensation. Figure 3 shows that the TAEC was above EI in 5 participants while ration size became rapidly insufficient in 4. From a practical point of view, it highlights the military challenge to elaborate fit‐for‐all menus, avoiding too many unconsumed items that would increase carried load, and providing portions large enough so that few or no soldiers feel limited. Finally, as in our previous studies, this variability in EI ranged on a continuum (from 69% to 99% of TAEC) and there was no possibility to separate "responders" and "non responders" as some authors have proposed (Finlayson et al., 2009; Finlayson et al., 2011). Interestingly, in their notebooks several participants reported hunger feelings, and added comments suggesting there was not enough available food during the expedition. Actually, from D10, three of them mentioned “Asleep with hunger feelings”, “Hunger feelings, not enough to eat! I am HUNGRY!” or “I fell asleep with hunger sensations, the portions are insufficient”. Conversely, one mentioned “Since the amounts of food are too much for me, I realized that I often ate out of self‐indulgence.” The increase in mean hunger scores may reflect some undetermined difficulties to increase their EI accordingly despite the possibility to eat additional foods from other participants.

Our results showed that the only increase in EI occurred between 10:00 and 14:00, that is, during the earlier diurnal nomad period. Although hunger scores were found to be increased on the morning of D11–15, no change in EI was observed between 06:00 and 10:00, when participants ate in the tent. Foods eaten during these sedentary periods may have been too similar (mostly lyophilized items) triggering a sensory specific satiety effect (Rolls, 1986) and the well described reduced motivation to eat. Moreover the size of the portions may not have been large enough and limited intake. Thus, it was probably easier for participants to increase EI during the diurnal nomad periods, since there was more variety in the available food items. This increased intake was found for the first (10:00–14:00) but not the second (14:00–18:00) nomad period, as it would have been expected with a strictly responsive energy compensation. This may result from an anticipatory compensatory behavior. This phenomenon can be cognitive, for example when a high‐EE exercise (Barutcu, Taylor, Mcleod, Witcomb, & James, 2020) or a 24‐hr severe energy restriction (James, James, & Clayton, 2020) is planned, leading subjects to increase intake at the prior meal or in the prior 24 hr, respectively, or conditioned, such as in humans omitting their habitual afternoon meal during 1 month, with a progressive increased EI at lunch (Chapelot et al., 2006). Even if we have no direct evidence in support of this hypothesis, this is consistent with the well‐known superior adaptive power of anticipatory behaviors in energy homeostasis (Chapelot & Charlot, 2019). This should be considered when preparing food for such expeditions, allowing individuals to increase intake prior to their high‐EE tasks. Further research specifically designed for assessing this important mechanism of eating behavior is required.

The only significant change that we observed in the food reward assessment was the increased preference for sweet foods during the D11–15 period. This result does not appear to be the consequence of an alpha risk, as a similar increased preference approached significance (*p = *.09) in our previous study (Charlot et al., 2020). Moreover two participants clearly verbalized this specific craving from D10: “I have a real craving for chocolate and sugar” and “I want sugar!”. Comparisons are difficult, as the LFPQ and the modified FPQ‐S_16_ have rarely been used in this field of research (Aeberli et al., 2013; Karl et al., 2018). However, historical studies in the Antarctic reported a sudden and rapid desire for fats (Frazier, 1945; Morton, 1997; Siple, 1945) even in individuals who had a previous abhorrence of fats (Frazier, 1945). Increased preference for sweet foods has only been reported during expeditions in high‐altitude (Aeberli et al., 2013; Pugh, 2004) that are sometimes characterized by a maintenance of carbohydrate intake despite large reductions in EI (Westerterp‐Plantenga et al., 1999). Carbohydrates are thought to partly alleviate respiratory burden through an increase in arterial saturation (Charlot, Pichon, Richalet, & Chapelot, 2013). However, since our expedition was not situated at altitude, this physiological mechanism is not relevant. Although preferences for food in questionnaires were not associated with the actual food choice at altitude (Aeberli et al., 2013), FP for sweet foods and carbohydrate intake were both higher in our present and previous results (Charlot et al., 2020), but not correlated. Moreover in our previous study (Charlot et al., 2020), some significant associations between EL scores and anthropometric (BM loss) or intake (EI) outcomes were found, suggesting that the LFPQ or modified FPQ‐S_16_ could potentially predict changes in anthropometry and/or EI. Further research is therefore needed to clearly determine the role of these questionnaires in predicting energy homeostasis in field studies.

Naturally, these results are limited to the specific and extreme conditions of such expeditions and may not be relevant to more ecological environments or in other populations. Another limitation is the distance between the researchers and the participants, most of the acquired parameters being based on trust. As a consequence, we were unable to use gold‐standard assessments of EI (derived from direct weighing of the unconsumed items by experimenter after meals), body composition (dual‐energy x‐ray absorptiometry), and EE (doubly labeled water). However, as described above, we developed solutions and tried our best to use valid technical surrogates to ensure a high level of accuracy in the data collection and all results show good within‐study consistency. For example, wrist‐worn optical HR monitors, used to assess EE, have been shown to provide reliable measurements of HR relative to traditional chest belts (concordance correlation coefficient >0.80 [Gillinov et al., 2017]). Moreover participants conserved the wrapping of both fully and partially consumed items, facilitating the recollection of the consumed amounts. As the composition and weight were known for all of the food by the researchers, the a posteriori determination of energy and macronutrient intake was the most accurate possible under these conditions. Concerning fluids, the use of thermos bottles and cups of known volume facilitated estimation of the consumed volumes. In addition, our participants were soldiers and highly implicated in every scientific survey aiming to improve their well‐being, skills, and operationality. Thus, their commitment and compliance were very high. Finally as already explained, the conditions of this expedition were particular and considered to be comfortable relative to other military expeditions/training (low operational strain, no sleep deprivation, and sufficient time to cook and eat). We concede that these differences weaken comparisons with most of these field studies. However, in addition to assessing several outcomes related to energy compensation kinetics in a field study in an adverse environment, this study offers exploitable leads for commands seeking to reduce energy deficiency of their soldiers (use a large selection of easy‐to‐use, highly palatable and familiar foods and allocation, as far as possible, of several long periods to eat).

## 5 CONCLUSIONS

This study shows that with an appropriate food supply, a thorough conception of rations, and a planned schedule stimulating spontaneous intake, energy homeostasis occurs even in extreme conditions, such as a 15‐day expedition in a cold environment with constant physical activity. A neutral EB was reached in <10 days and participants were no longer in low EA after 10 days through a compensatory increase in EI with some indications of an anticipatory mechanism. Contrary to our previous report, few changes in food reward were observed except some increased preference for sweet foods. Future expeditions will require in‐depth exploratory subjective and objective measures to improve our knowledge about the hedonic, metabolic and environmental determinants of energy homeostasis.

## CONFLICT OF INTEREST

No potential conflicts of interest relevant to this article were reported.

## AUTHORS’ CONTRIBUTION

KC, PC, and CB designed the study. KC, JS, CL, PC, and CB prepared the expedition. KC and CL collected and processed data and conducted the statistical analyses. Laboratoire graphique© (KC) designed the figures. KC drafted the initial manuscript. DC wrote the second draft of the manuscript. All authors reviewed and revised the manuscript, approved the final manuscript as submitted, and agree to be accountable for all aspects of the work.

## ETHICAL STATEMENT

This study was performed at the request of the French Armée de Terre and approved by the scientific leadership of the French Armed Forces Biomedical Research Institute. This study required no invasive measurements and did not impose unfamiliar tasks to the participants. In this case, we were exempted according to the Institute regulation to obtain an ethical approval from a civilian Committee as long as the experiment was realized in accordance with the Declaration of Helsinki.

## Data Availability

The datasets generated and analyzed during the current study are available from the corresponding author upon reasonable request.

## APPENDIX A

**Table:**

Product denomination	Brand	Number of apparition(s)
**Lyophilized breakfast sweet dish ×1 (105 ± 9 g to 1,654 ± 298 kJ)**		
Milk and red fruits muesli		5
Super seed & red berry muesli	*Tentmeals*	1
Blueberry burst breakfast	*Tentmeals*	1
**Lyophilized breakfast savory dish ×1 (109 ± 25 g to 2,010 ± 120 kJ)**		
Posh pork and beans	*Firepot*	3
Scrambled eggs with cheese	*Summit to eat*	2
Scrambled eggs	*Adventure food*	2
**Chocolate sandwich ×1 (60 g to 502 kJ)**	*Bridgford*	7
**Fruit jellies ×2 (60 ± 0 g to 399 ± 6 kJ)**		
Apricot flavor	*Pierrot Gourmand*	3
Pear flavor	*Pierrot Gourmand*	4
Raspberry flavor	*Pierrot Gourmand*	4
Red fruits flavor	*Pierrot Gourmand*	3
**Energy gels ×1 (20 ± 0 g to 252 ± 8 kJ)**		
“Antioxidant”	*Meltonic*	3
“Endurance”	*Meltonic*	2
“Boost”	*Meltonic*	2
**Dark chocolate bar ×1 (25 g to 1,015 kJ)**	*Klaus*	7
**Energy bars ×1 or ×2 (89 ± 10 g to 1,753 ± 71 kJ)**		
Coconut bars **×**2	*Rapunzel*	2
Cranberry almond bar	*Be‐Kind*	3
Almond and cashew origin bar	*Overstim's*	2
Nut bar	*Fuzion*	1
Almond punchy bar ×2	*Punch power*	2
Banana punchy bar ×2	*Punch power*	1
**Sweet bars ×1 (45 ± 7 g to 968 ± 133 kJ)**		
Balisto almond bar	*Balisto*	1
Kit kat chunky	*Kit Kat*	1
Snickers	*Snickers*	1
Lion	*Lion*	1
M&Ms	*M&Ms*	1
Bounty ×2	*Bounty*	1
Kinder Country ×2	*Kinder*	1
**Jerky beef (35 g to 434 kJ)**	*Feed & Go*	**7**
**Tortilla ×1 (40 g to 514 kJ)**	*Old El Paso*	7
**Cheese ×1 (40 ± 0 g to 645 ± 51 kJ)**		
Emmental	*Auchan*	1
Comté	*Auchan*	1
Gouda	*Auchan*	1
Meule fruitée	*Auchan*	1
Massdam	*Auchan*	1
Parmiggiano	*Auchan*	1
Leerdamer	*Leerdamer*	1
**Saucisson ×1 (29 ± 2 g to 610 ± 58 kJ)**		
Sticks”nature” ×3	*Auvernou*	1
Sticks “emmental” ×3	*Auvernou*	1
Sticks “roquefort” ×3	*Auvernou*	1
Sticks “goat cheese” ×3	*Auvernou*	1
Chorizo sticks ×3	*Auvernou*	1
Sticks”nature” ×3	*Auchan*	1
Mini‐saucisson “nature” ×4	*Auchan*	1
**Lyophilized beef (20 g to 1,295 kJ)**	*Marie de Livinhac*	7
**Lyophilize soup (18 ± 1 g to 275 ± 73 kJ)**		
Leek cream	*Titok*	1
Oriental potage	*Neff*	1
Mushroom soup	*Maggi*	1
Tomato soup	*Maggi*	1
8‐vegetable potage	*Maggi*	1
Hen bouillon	*Maggi*	1
Asparagus cream	*Maggi*	1
**Lyophilized diner savory dish ×2 (264 ± 16 g to 4,725 ± 245 kJ)**		
French Aligot	*MX3*	1
Lentils with ham	*MX3*	1
French Tartiflette	*MX3*	1
Royal Paella & saffron	*MX3*	1
Oriental chicken Tajine	*MX3*	1
Korma chicken & rice	*MX3*	1
French Truffade	*MX3*	1
Beef stew & pasta	*MX3*	1
Chicken mushroom pasta	*MX3*	1
Dauphinoise potatoes	*Voyager*	1
Beef mushrooms & rice	*Voyager*	1
Chicken couscous	*Voyager*	1
Cheese‐topped potatoes with smoked ham	*Voyager*	1
Chili con carne	*Real Turmat*	1
**Lyophilized dessert ×1 (95 ± 11 g to 1,718 ± 120 kJ)**		
Custard apple crunch	*Fuel your Preparation*	1
Chocolate mousse with cherry & granola	*Summit to Eat*	1
Chocolate mousse	*Trek’N’Eat*	1
Instant dessert with raspberries	*Travellunch*	1
Rice pudding with apples and cinnamon	*Travellunch*	1
Strawberry cream cheese	*Travellunch*	1
Wild berry yogurt dessert	*Travellunch*	1

In bold are presented the items included in each of the seven menus with the mean ± *SD* mass and energy content. Below are presented the denomination of the different recipes/tastes of each item, their respective brands and the number of time they were presented in the seven different menus.
